# Paediatric Inflammatory Bowel Disease: A Mechanistic Approach to Investigate Exclusive Enteral Nutrition Treatment

**DOI:** 10.1155/2014/423817

**Published:** 2014-05-21

**Authors:** Lily Nahidi, Andrew S. Day, Daniel A. Lemberg, Steven T. Leach

**Affiliations:** ^1^School of Women's and Children's Health, University of New South Wales, Randwick, Sydney, NSW 2052, Australia; ^2^Department of Paediatrics, University of Otago, Christchurch, Christchurch 8140, New Zealand; ^3^Department of Gastroenterology, Sydney Children's Hospital, Randwick, Sydney, NSW 2031, Australia; ^4^Westfield Research Laboratories, Level 2, Sydney Children's Hospital, High Street, Randwick, NSW 2031, Australia

## Abstract

The inflammatory bowel diseases (IBD) include Crohn's disease (CD) and ulcerative colitis. The disease may present at any age although the peak of presentation is the second and third decades of life. The incidences of these diseases are increasing around the world with the age of presentation getting younger. At present CD is incurable with colectomy being the treatment for severe UC. Although several pharmacological approaches are used to modulate the inflammatory response in IBD, few lead to histological healing and most have side effects. An alternative approach is to use enteral formulae given exclusively (EEN) to treat IBD. EEN requires the consumption of an elemental or polymeric formula, with the exclusion of all other nutrients, for a period of up to 12 weeks. The introduction of EEN as a therapeutic option for IBD was through prudent observation; however, EEN has become an established and reliable option for the treatment of paediatric IBD. Despite this, the mechanisms through which EEN induces disease remission are unknown and remain hypothetical. This review will discuss recent research into EEN both describing clinical features of EEN therapy and discussing the most up-to-date understanding of the mechanisms through which EEN may be reducing intestinal inflammation and inducing disease remission.

## 1. Introduction

Inflammatory bowel disease (IBD) is a chronic affliction predominantly involving the gastrointestinal tract and includes both Crohn's disease (CD) and ulcerative colitis (UC). Although IBD may present at any age, the peak period of presentation for CD and UC is the second and third decades of life, especially in adolescence [1, 2]. Over recent decades, an increasing incidence of IBD, particularly of CD, has been reported around the world [1–6]. In addition to the increasing frequency of diagnosis, it is also reported that CD now presents at earlier ages [2].

At present CD is incurable with colectomy being the treatment for severe UC. Therefore current therapeutic options serve to control inflammatory flares and reduce adverse outcomes [7]. Current pharmacological therapies include antibiotics, corticosteroids, aminosalicylates, and immunosuppressive agents. However, for disease beginning in childhood, there is a desire to reduce and/or eliminate the reliance upon pharmacological therapies for disease control, especially given the many decades of disease that will follow. Therefore exclusive enteral nutrition (EEN) is increasingly becoming an established option for the treatment of paediatric IBD. This review will discuss recent research into EEN as well as discussing potential mechanisms of action of this therapy.

## 2. Clinical Features, Aetiology, and Pathogenesis of Paediatric IBD

IBD is characterised by inflammation of the bowel. Inflammation in CD may involve any part of the GI tract and is characterised by discontinuous and transmural inflammatory lesions [2, 7–12]. The most common areas affected by CD are the ileum and colon [2, 7–12]. In addition, CD is associated with a variety of extraintestinal manifestations [13–15]. In UC, however, the inflammatory changes are considered to only involve the colon, with involvement of the superficial layers of the intestinal mucosa [7–12, 16–20].

The common characteristic clinical presentations of CD include recurrent abdominal pain and severe diarrhoea [2, 7–10, 21, 22]. The presentation in CD is usually subtle, often leading to a delayed diagnosis [7–10, 23]. Inflammatory markers used to diagnose IBD are listed in Table 1. The prominent presenting features of UC are severe diarrhoea with blood and mucus mixed in stool accompanied by lower abdominal cramping [1, 7–10, 16–18]. Due to the presence of blood and/or mucus in the stools, UC is often diagnosed earlier following the onset of symptoms compared to CD [14]. Patients with IBD may also experience a number of extraintestinal manifestations that may produce greater morbidity than the underlying intestinal disease [13, 15]. Further complications of IBD can also include weight loss, delayed growth and pubertal development in children, nutritional deficiencies, and bone abnormalities (e.g., osteopenia and development of osteoporosis).

Considerable progress has been made in IBD research, there is no adequate explanation for the exact aetiology of IBD, although it is considered multifactorial [11, 24–29]. Defective intestinal epithelial barrier, environmental risk factors, bacterial pathogenesis, and/or bacterial byproducts in combination with susceptible genetics cause temporary or permanent tissue injury to the intestine in the form of inflammation and ulceration [24–28]. Like most chronic inflammatory diseases, IBD has been primarily regarded as a disease of industrialised and Western culture, with increased prevalence in developed countries [8–10]. Furthermore, developing countries historically reported lower prevalence of IBD, although with increased industrialisation there is now increasing incidence of both CD and UC [8–10]. Hence, increasing incidence of IBD over the last half-century suggests changes in the environment as contributing factors in the aetiology and pathogenesis of IBD [8–10]. So far, a number of theories for environmental causes of the disease have been postulated, and some environmental risk factors for IBD have been identified [8–10].

The best documented environmental lifestyle association to date is tobacco use, particularly cigarette smoking [8, 16–18, 30]. Notably, cigarette smoking has an opposite impact on CD and UC [8, 16–18, 30–37]. Independent studies have demonstrated that UC is predominantly a disease of nonsmokers and ex-smokers, supporting a protective role of smoking in this disease [16, 30–37]. Contrary to findings in UC, there is strong evidence supporting a 2–4-fold increased risk of development of CD with smoking [16, 30–37].

Another potential environmental risk factor is stress. Since at least the 1930s there has been debate about the contribution of emotional stress, anxiety, and tension to the inflammatory process in both forms of IBD without any definitive resolution [8, 10]. Recent human and animal studies provide compelling evidence that stress can affect the GI tract in a number of ways, including alteration of intestinal permeability [8, 10] and mucosal proinflammatory cytokines production [8, 10].

However smoking and stress are unlikely to have prominent roles in the aetiology of paediatric IBD and it is likely that diet and nutrition play larger roles in this age group. Considerable research has been undertaken to look for possible links between dietary components and IBD pathophysiology. Rising rates of IBD, both CD and UC, in developing countries with a “westernisation of diet” suggest that preillness diet could be a risk factor for the pathogenesis of both CD and UC [8, 21, 38–41]. During the mid-20th century the dietary pattern and physical activities in many countries changed with highly processed food coming into widespread use [8, 21, 38–41].

A number of dietary studies have been carried out to examine whether increased consumption of carbohydrate, fat, fruits, and fast foods is associated with an increased risk of IBD [8, 21, 38–41]. The evidence across different studies consistently shows that dietary intake of fat, meat, fast foods, and sugar contributes significantly to the development of IBD [8, 10, 38–40]. In line with this view, Persson et al. [42] have ascertained that there is a positive association between the intake of fast foods and the incidence of IBD in young and middle-aged adults [42]. Although not investigated directly, the composition of the fat intake (n-3 and n-6 fatty acids), which are metabolised to immunomodulatory leukotrienes and prostaglandins, might provide an explanation for the increased risk of IBD [8, 10, 38–40, 43–46]. In addition, an epidemiological study in Japan reported an increased consumption of meat, animal protein, and diet rich in fat, closely correlated with a higher risk of the disease in individuals [43–46]. A further Japanese study reported more than 2-fold increased risk of CD following consumption of chocolate, sweets, and coca drinks, with high sugar content, fat, and oil [45]. Similar associations have also been reported in the paediatric population in Canada [47].

Conversely there is also evidence of dietary factors that are protective against IBD including daily vegetable consumption and whole meal bread consumption [48]. In addition a systematic review of the literature on breastfeeding and the development of paediatric IBD has shown that breastfeeding has an overall protective effect on developing early-onset IBD [49]. However, the authors point out that the currently available literature is generally of poor quality and there is a need for well-designed prospective studies to sufficiently investigate this question [49].

A significant proportion of the research effort into IBD has been directed towards the genetic component of the disease. Greater than 150 susceptibility loci have been identified to date and more than 70% of these loci are shared with other inflammatory diseases [50]. The strongest association for CD is seen with several risk alleles in the* CARD 15* (*NOD2*) gene: studies indicate up to a 20-fold or more increased risk of developing CD when such risk alleles are present [51]. The CARD15 protein recognises muramyl dipeptide (MDP), a bacterial cell wall component derived from peptidoglycan [52]. MDP is transported to cytoplasmic CARD15 with subsequent activation of the transcription factor, NF-*κ*B, thereby initiating production of numerous inflammatory mediators [53]. CARD15 is a negative regulator of Toll-like receptor 2- (TLR2-) mediated Th1 responses [25] and mutated CARD15 is associated with diminished mucosal defensin production [54].

### 2.1. Bone Loss and Development of Osteoporosis in IBD

Bone is a dynamic tissue that is continually remodelled throughout life, consequent to the opposing activities of osteoclasts and osteoblasts [55–57]. During the remodelling cycle, bone generation by osteoblasts is coupled to bone resorption by osteoclasts [55–58]. The equilibrium of osteoclasts/osteoblasts and therefore bone remodelling is principally coordinated by 3 different soluble inflammation-associated mediators. These mediators are the receptor activator of NF-*κ*B (RANK), its ligand (RANK-L), and osteoprotegerin (OPG), which are members of the TNF and TNF-receptor superfamilies [55–61]. In the bone microenvironment, RANKL and its soluble form (sRANKL) are expressed on the surface of osteoclasts, B cells, activated T cells, and stromal cells, while OPG (a decoy receptor for RANK) is produced by different tissues and cell types including osteoblasts, B cells, dendritic cells, and bone narrow stromal cells [56–59, 61]. The binding of RANKL to its physiological receptor RANK induces a cascade of signalling leading to differentiation and maturation of osteoclast [55–58, 61]. However, OPG interferes with the bindings of RANK/RANKL and retards this signalling [55–59, 61]. Therefore, an imbalance between OPG and RANKL can alter rates of osteoclastogenesis and can result in altered bone loss.

Poor bone acquisition and increased fracture risk are significant complications in paediatric IBD [62, 63] and multiple cytokines including TNF-*α*, IL-6, IL-8, and IL-1, elevated in IBD, are also associated with increased risk of bone fractures [55, 58–61, 64, 65]. Therefore it is likely that inflammation could contribute to the development of osteoporosis through RANK/RANKL/OPG system [55–59, 61]. A number of studies have shown disturbances to the RANK/RANKL/OPG system in CD patients with altered serum OPG [59, 60, 64] and RANK expression in tissue from CD patients, who had decreased bone mineral density and reduced markers of bone formation [63, 66–69]. In addition, in CD patients, an imbalance between OPG and RANKL may represent a continuing homeostatic response attempting to reverse established osteopenia and RANKL-driven osteoclastogenesis, thus maintaining normal bone mass [55–61, 66]. Further to this we have identified that mucosal and faecal OPG is elevated in active paediatric IBD [70] and have recently shown that OPG possesses proinflammatory properties and may contribute to the mucosal inflammatory response in IBD [71]. Although, OPG and RANKL likely participate in a complex cytokine network that regulates numerous functions in the immune system and bone maintenance, further studies are still required to advance our understanding of their interactions in IBD pathogenesis.

## 3. IBD Treatments

IBD is characterised by a variable chronic relapsing course, in which periods of disease control are interrupted by periods of increased disease activity [7, 9, 10, 25]. When choosing the best management strategy for IBD a number of variables must be considered including the location and disease severity, extraintestinal symptoms, complications, and the response to previous treatment [7, 72, 73]. Current pharmacological therapies comprise those used to achieve remission and those used to maintain remission [7, 72–76]. Previously, the goal of therapy was solely to reduce inflammation, and although this is still a priority, achieving mucosal healing is now considered the gold standard of therapy. However, while current pharmacological therapies can rapidly alleviate symptoms, they do not cure IBD and lead to limited improvements in mucosal inflammatory lesions and mucosal healing, with the exception of infliximab [74]. Furthermore, in the setting of paediatric disease the use of many of the current pharmacological agents can be associated with undesirable side effects consequent to prolonged use [77]. An alternative approach to the management of IBD, mainly CD, is EEN, a therapeutic option that was first introduced 3 decades ago and which has been shown to be a valid therapeutic option in adults and children [78, 79].

EEN involves the provision of a liquid diet using elemental or polymeric formula (PF), given exclusively over a prolonged period of up to 12 weeks. Elemental formula contains individual amino acids, while PF is composed of intact proteins. Paediatric studies have demonstrated that PF has equivalent efficacy to elemental formula, but due to their improved palatability, PF are also associated with increased tolerance and compliance [72, 80, 81]. Meta-analysis of paediatric studies has shown that EEN possesses equivalent efficacy to corticosteroids in the induction of remission [77]. In addition to reducing mucosal inflammation, EEN leads to superior mucosal healing and nutritional improvements, has few side effects, and permits avoidance of medication-related side effects [72, 73, 82, 83].

EEN has been shown to induce remission in approximately 85% of paediatric patients with CD [72, 73, 84]. At the Sydney Children's Hospital, Australia, induction of remission with EEN as sole therapy in 80% of a group of children with newly diagnosed CD has been demonstrated [84]. In these children, falling disease activity (mean PCDAI decreasing from 37.1 ± 10.8 to 6.7 ± 5.1) and decreasing inflammatory markers (including CRP) after 8 weeks of therapy were noted [84]. These improvements were seen in children regardless of the location of their disease, including those with isolated colonic disease. Furthermore, these children had mean weight gain of 4.7 ± 3.5 kg [84]. In a subsequent cohort of 17 children managed with EEN, it was also demonstrated that 8 weeks of EEN reduces levels of carboxy-terminal collagen crosslinks (CTX), a marker of bone resorption, from 2.2 ± 0.4 to 1.6 ± 0.4 ng/mL (*P* < 0.01) whilst bone formation markers increased [85]. The combination of reduced inflammation and improved nutrition has been suggested as the mechanism by which EEN normalises markers of bone resorption and formation [72, 73, 86]. However it is also intriguing that EEN treatment normalised intestinal OPG levels, which may in part, be due to direct suppression of OPG/TNF-*α* production in intestinal epithelial cells [70]. Considering that OPG participates in the complex network of inflammatory processes and connects gut inflammation to systemic events, regulation of OPG by EEN in the intestinal mucosa may also contribute to improved bone health.

An additional feature of EEN therapy is the correction of micronutrient deficiencies such as vitamin D deficiency. Levin et al. reported that vitamin D levels were high in children with IBD following initial treatment with EEN compared to children who were initially treated with corticosteroids [87]. Analysis of the two groups indicated that disease characteristics between the two treatment groups were very similar, and in the absence of cotherapy, the increased vitamin D levels were attributed to the EEN treatment [87]. There is now substantial evidence indicating that vitamin D has a role in immune regulation. In the setting of IBD the role of vitamin D in disease pathogenesis requires further investigation; however, initial findings show that vitamin D sufficiency can be beneficial. Jorgensen et al. reported on that vitamin D supplementation significantly reduced the risk of relapse in CD [88].

There have been several proposed mechanisms by which EEN may reduce inflammation. One early suggestion was through gut rest. However as both elemental and polymeric formulas show similar results, gut rest is unlikely to be the primary factor in reducing inflammation. Additional proposed mechanisms include normalisation of altered permeability, direct modification of mucosal responses, and alterations in intestinal bacteria.

### 3.1. Altered Permeability

Defects in intestinal epithelial tight junction barrier function have been demonstrated to lead to increased intestinal permeation to toxic luminal substances (e.g., bacteria, bacterial by products, antigens, and enzymes). Altered permeability has been reported in those suffering from CD [24, 26, 27]. Several studies have ascertained that CD patients have an abnormal increase in intestinal permeability to paracellular markers, such as polyethylene glycol 400, cellobiose, Cr-ethylenediaminetetraacetic acid (Cr-EDTA), and lactulose [24, 26, 27]. Further studies have also shown that healthy first-degree relatives of CD patients have increased intestinal permeability with evidence of antigenic stimulation [89]. Based on these observations, it has been suggested that the defective gut barrier function could be a primary aetiological factor of CD [9, 23, 24, 26, 27].

We have investigated the effects of EEN therapy upon intestinal permeability in both an* in vitro* system and in a mouse model of colitis. Our* in vitro* model consisted of immortalised human colonic epithelial cells grown to confluence on a membrane support, with the epithelial monolayer separating upper (luminal) and lower (basal) compartments. This system allows the monolayer to be exposed to PF in the luminal compartment and TNF in the basal compartment. Initial investigations using this model found that PF exerted an anti-inflammatory effect by acting directly upon epithelial cells to reduce release of the proinflammatory chemokine interleukin-8 (IL-8) [90]. Further investigation also revealed that exposure to PF was able to maintain normal transepithelial electrical resistance, short circuit current, paracellular permeability, and morphological distribution of tight junction proteins [91] and therefore maintain normal permeability and gut barrier function. This restoration of gut barrier morphology and function by PF was principally associated with a mechanism involving inhibition of MLCK [91].

These* in vitro* findings were then further supported by studies utilising an IL-10 KO mouse model of colitis [92]. In this model, colitis was initiated by exposure to a species of* Helicobacter*. Exposure to the bacteria resulted in progressive weight loss, increased gut inflammatory markers, and reduced gut barrier function [92]. However, mice exposed to* Helicobacter* sp. and receiving EEN treatment were found to have normal gut barrier function and maintained gut barrier integrity, reversed inflammatory changes along with reduction of bacterial load [92]. These findings suggest that EEN possesses anti-inflammatory properties as well as antibacterial properties.

### 3.2. Direct Anti-Inflammatory Properties of Polymeric Formula

Clinical studies have also shown decreased proinflammatory mediators in response to EEN [93], with increased mucosal levels of protective proteins such as TGF-*β* [82]. There is developing evidence that enteral feeds have direct anti-inflammatory effects upon epithelial cells [90, 94]. For example, Meister et al. [94] demonstrated that elemental feeds have direct anti-inflammatory effects upon the gastrointestinal mucosa. In their studies, endoscopically obtained mucosal biopsies from adults with CD, UC, and control subjects were incubated with elemental formula for up to 24 hours. Incubation of tissues from patients with CD lead to an increased ratio of IL-1Ra to IL-1*β* compared to the ratio in control samples (*P* < 0.05) [94]. Changes in this cytokine ratio were not observed in biopsy samples from individuals with UC or noninflammatory controls [94]. These results correspond to* in vivo* human data showing changes in mucosal anti-inflammatory and proinflammatory proteins as a consequence of EEN.

Using our* in vitro* model to further clarify the effects of enteral feeds upon epithelial cells [90], preliminary data indicates that PF reduces colonic epithelial cell chemokine responses to the proinflammatory cytokine TNF-*α* [90]. Specifically, our results showed that pretreatment of HT-29 colonic epithelial cells with a PF decreased cellular IL-8 production by TNF-*α* stimulation from 21,224 ± 2820 pg/mL to 2333 ± 828 pg/mL (*P* < 0.01) [90]. These results suggested that direct intracellular mechanism was involved. Further, we investigated the effect of PF on cytoplasmic I*κ*B and observed that degradation of I*κ*B in response to TNF was both delayed and reduced when cells were exposed to PF [90]. Therefore, we have proposed that PF directly interact with components of the NF-*κ*B pathway [90].

In further experiments utilizing this* in vitro* model we have recently identified that PF can further alter immune responses through reduction of TNF-*α*-induced expression of ICAM-1 to suppress production and/or circulation of proinflammatory cytokines including IL-1, -6, -8 and TNF-*α* (unpublished observations). Furthermore, we have observed that PF modulates NF-*κ*B activity not only by increasing inhibitors of NF-*κ*B and inhibiting the kinase activity of NF-*κ*B subunit, but also by reducing the signal transduction from members of TNF receptor superfamily, which, in part, limits the NF-*κ*B self-stimulation loop. We have also observed that PF also corrects proinflammatory-induced intestinal defects though modulation of ROCK, MPRIP, and MYLK2 genes (unpublished observations). These findings implicate that PF is directly altering the response of epithelial cells to proinflammatory stimuli. The resulting net effect is that epithelial cells have a substantially reduced response to these stimuli.

### 3.3. Alteration of Gut Microbiology

Initial investigation of EEN indicated profound alterations in the bacterial diversity of mucosal biopsies after EEN treatment compared with biopsies collected prior to EEN treatment [95]. The authors used PCR-denaturing gradient gel electrophoresis (PCR-denaturing gradient gel electrophoresis (DGGE)) with universal bacterial primers (Eubacteria) to compare the intestinal mucosal flora of 6 children with CD prior to and following treatment with EEN [95]. This study showed that marked changes in bacterial diversity were present in the posttreatment intestinal biopsies as compared with that collected at baseline (prior to EEN). A similar result has been reported by Lionetti and investigators [96] who also observed, using a similar technique with universal bacterial primers, that during and following treatment with PF, the diversity and distribution of intestinal bacteria in serial mucosal biopsies from 10 children with CD differed profoundly from that at baseline.

Consistent with this evidence are studies from our own group that clearly demonstrated altered faecal microflora patterns in children with newly diagnosed CD within a week following commencement of EEN and that these changes persisted throughout the 8 weeks of therapy [97]. In this study, primer sets for the 5 major bacterial groups (providing 90% coverage of intestinal bacteria) present in the intestine were included [97]. The primer sets included Eubacteria, Bacteroides-Prevotella group, the* Clostridium coccoides* group, the* Clostridium leptum* subgroup, and the Bifidobacteria group [97] with serial stool samples (baseline (prior to EEN), 1, 2, 4, 6, and 8 weeks (during EEN treatment), and 16 and 32 weeks (after EEN)) collected from 6 children with CD. Examination of the DGGE profiles showed that during and following EEN treatment the intestinal flora was significantly altered as compared with the baseline samples [97]. Within a week after starting EEN, bacterial diversity was reduced and there was evidence of changing bacterial composition that persisted throughout EEN treatment [97]. Following resumption of normal diet there was recovery of bacterial diversity; however, there were dramatic changes to bacterial composition, as indicated by DGGE profiles, evident four months after therapy [97]. A further interesting finding was that change in the Bacteroides-Prevotella group of bacteria was significantly correlated with reduction in disease activity [97].

It is unclear what factors may be driving the alterations in gut bacteria in EEN. There are a number of possibilities including influence of mucosal and epithelial events affecting intestinal bacteria composition via crosstalk, possible prebiotic properties of the formulas used for EEN, and possible limitation of bacterial access to energy and/or nutrients during EEN. Further investigation using next generation sequencing techniques is underway to more precisely define changes in the bacterial community during EEN therapy. However it is clear that further research into how nutritional therapies, such as EEN, alter intestinal bacterial composition is required. A more intimate understanding of this area will undoubtedly assist in treating IBD and other diseases.

### 3.4. A Mechanistic Model Approach

When considering all these findings we hypothesize that a mechanistic model approach can be used to explain the clinical activity of EEN. PF has been shown to have activity in different and distinct compartments that both contribute to IBD pathogenesis. The first compartment is the epithelial cells. Although epithelial cells are not the only cellular source of inflammatory stimulus, they are the most prominent cells in controlling the interaction and exposure of the underlying mucosa to the luminal environment. PF has been shown to both reduce the release of proinflammatory cytokines from these cells and to maintain gut barrier function, therefore limiting exposure of the underlying lamina propria to the gut luminal environment. We propose that it is the improvement of gut barrier function that is intimately connected to resolution of the inflammatory process in the mucosa.

The second compartment of importance is the intestinal microflora. Although the precise mechanism by which PF/EEN influences the intestinal microflora is unknown, there is sufficient evidence to conclude that the course of EEN changes the intestinal microflora resulting in a less “pathogenic” microflora composition. The activity of PF/EEN in both reducing the pathogenic potential of the luminal environment, limiting the exposure of the underlying lamina propria to the luminal environment, and reducing the proinflammatory response of epithelial cells to the luminal environment results in reduced disease activity. Currently, this mechanistic model has not yet been validated by specific experimental testing. Nevertheless we propose that this model can provide the framework for further studies to understand the actions of EEN in greater detail.

## 4. Conclusion—EEN: A Combination Therapy

The clinical benefits of EEN have been well defined. They include improvements in the nutritional status of patients, reduced disease activity scores, decreased mucosal proinflammatory cytokines, and increased mucosal levels of growth-related proteins (e.g., TGF-*β*). However the mechanisms of these effects remain unclear. Proposed putative mechanisms include relative bowel rest, alteration of the intestinal microflora, and direct anti-inflammatory effects.

The initial use of elemental feeds for EEN leads to the suggestion that gut rest could be important. Subsequently, polymeric feeds were shown to be equally beneficial [80, 81]. Consequently, gut rest alone would appear unlikely, as PF would be expected to require more digestive activity than elemental formula. Therefore, based on the available evidence, it is reasonable to consider that EEN prompts the observed clinical effects through restoration and maintenance of gut barrier function, suppression of intestinal inflammation, and modulation of the intestinal flora. And it is the likely combination of all of these three activities that contributes to EEN suppressing disease activity.

However, this may only be the tip of the iceberg in regard to nutritional therapy. We propose that further advances in this therapy can be achieved. Investigation of the interactions between NF-*κ*B-related genes whose expressions were modulated by PF could further advance our understanding of the pathogenesis of IBD and could also be potential therapeutic targets for future intervention. Investigation of the impacts of RANKL on gut barrier function, tight junction paracellular permeability, mucosal production of proinflammatory cytokine, and NF-*κ*B signalling pathway would be important to study the capacity of PF in modulation of RANKL expression. These studies have potential to lead to developments of further noninvasive marker for IBD. Finally, understanding the mechanisms of EEN will assist in the development of more effective therapies. Improvements in EEN therapy would help reduce the reliance upon the current pharmacological IBD drugs, which should improve the overall quality of life and long-term health outcomes for these patients.

## Figures and Tables

**Table 1:** Inflammatory markers used in the diagnosis of IBD.

Inflammatory markers	
C-reactive protein (CRP)	

Erythrocyte sedimentation rate (ESR)	

Leucocyte and platelet count	

Albumin Hematocrit	

Crohn's Disease Activity Index (CDAI) in adults and Paediatric Crohn's Disease Activity Index (PCDAI) in children^1^	

Faecal calprotectin	

Faecal S100 A12	

Fecal M2-pyruvate kinase (M2-PK)	

^1^CDAI/PCDAI consists of a number of subjective and objective measures to provide an overall indication on disease activity. The laboratory components for PCDAI are ESR, albumin, and haematocrit.
